# Genome-wide identification and expression divergence of the NDPK gene family in *Brassica juncea* under abiotic stress

**DOI:** 10.3389/fpls.2026.1917522

**Published:** 2026-08-06

**Authors:** Huachuan He, Zhengjia Li, Xiaomei Wang, Xuming Xie, Qinwen Lei, Jingdong Chen, Heping Wan, Xiangxiang Zhang, Hao Zhang

**Affiliations:** 1 College of Life Sciences, Jianghan University, Wuhan, China; 2 China-Cuba Joint Natural Products Research Laboratory/Wuhan (China-Cuba) Joint Laboratory for Natural Products under the Belt and Road Initiative, Wuhan, China; 3 Key Laboratory of Biology and Genetic Improvement of Oil Crops, Ministry of Agriculture, Oil Crops Research Institute, Chinese Academy of Agricultural Sciences, Wuhan, China

**Keywords:** abiotic stress, *B. juncea*, functional characterization, gene family, NDPKs

## Abstract

Nucleoside diphosphate kinases (NDPKs) constitute a multifunctional enzyme family that is widely distributed in prokaryotes and eukaryotes and plays important roles in energy metabolism, stress perception, signal transduction, and reactive oxygen species homeostasis. However, this gene family has not been systematically characterized in *Brassica juncea.* Here, we performed a genome-wide identification and comprehensive analysis of the *BjNDPK* gene family by integrating bioinformatic analyses with expression and physiological assays. Eighteen non-redundant *BjNDPK* genes were identified and classified into five phylogenetic groups with distinct structural features. Cis-acting element analysis showed that the promoters of several members contain drought-responsive elements. Collinearity analysis indicated complex subgenomic rearrangement and selective retention during the allotetraploid evolution of *B. juncea*. Expression profiling showed that many *BjNDPK* genes differed among tissues and responded to PEG-induced osmotic stress and salt stress. Reference-gene stability analysis identified *qBjActin* as the most stable reference gene under the tested conditions. Physiological assays showed that PEG treatment increased the content of H_2_O_2_ in *B. juncea* leaves, whereas an *Arabidopsis thaliana atndpk2* (corresponding to *BjNDPK7* in *B. juncea*) mutant maintained greater root elongation than the wild type under mannitol treatment. These findings describe the structural, evolutionary, and expression characteristics of the *BjNDPK* family and provide candidate genes for future functional studies of abiotic-stress responses in polyploid *B. juncea*.

## 1 Introduction

Plants frequently encounter various abiotic stresses during growth and development, such as salinity, drought, low temperatures, and high temperatures. These adverse environmental factors not only disrupt cellular homeostasis but also significantly reduce crop yield and quality, posing major challenges to sustainable agricultural development (Krasensky and Jonak, 2012; Zhu, 2016; Puppala et al., 2023). To cope with stress, plants have evolved a complex and finely tuned molecular regulatory network over long-term evolution. Relevant genes play crucial roles in stress perception, signal transduction, and response processes (Liu et al., 2025; Zou et al., 2025; Guo et al., 2026).

Nucleoside diphosphate kinase represents a multifunctional enzyme family widely distributed across both prokaryotic and eukaryotic organisms (Yu et al., 2017). NDPK proteins universally contain a highly conserved catalytic domain (PF00334) and exhibit substantial homology across different species (Ishikawa et al., 2003). Their classical function is to catalyze phosphate transfer between nucleoside triphosphates (NTPs) and nucleoside diphosphates (NDPs), thereby maintaining intracellular nucleotide homeostasis and energy metabolism (Yu et al., 2017). Recent studies reveal that NDPKs not only participate in basal metabolism but also play vital roles in cellular signaling, antioxidant regulation, and stress adaptation, making them indispensable factors for sustaining normal cellular physiological activities (Dorion et al., 2006b).

Plant NDPKs are of particular interest because they link a conserved metabolic function with multiple stress-related regulatory processes. Unlike many stress-associated gene families whose members primarily function as transcription factors, transporters, or antioxidant enzymes, NDPKs catalyze phosphate transfer between nucleoside diphosphates and triphosphates and thereby contribute to cellular nucleotide and energy homeostasis. Individual plant NDPKs have also been implicated in ROS regulation, hormone signaling, photosynthetic processes, root development, and responses to salinity, drought, cold, and oxidative stress. Functional studies in *A. thaliana* (Kim et al., 2011; Ovečka et al., 2014; An et al., 2025), rice (Ye et al., 2020), maize (Kopylov et al., 2015), and several other plant species have demonstrated that the roles of NDPKs vary among family members and cellular compartments (Escobar Galvis et al., 1999; Dorion et al., 2006a; Valenti et al., 2007; Wang et al., 2024). These dual metabolic and regulatory properties make *NDPK*s relevant candidates for investigating how cellular energy status is coordinated with plant stress responses.

*B. juncea* is a vital oilseed, vegetable, and forage crop extensively cultivated in China, India, and Southeast Asia, playing a significant role in global food and oil production and multipurpose utilization. However, its growth and yield are highly susceptible to abiotic stresses such as salinity and drought, posing severe challenges to breeding and industrial development. Therefore, identifying key gene families closely associated with stress responses in *B. juncea* is crucial for understanding molecular regulatory mechanisms and advancing stress-tolerant breeding. However, the NDPK family has not been systematically characterized in this species. Specifically, the number and evolutionary relationships of *BjNDPK* genes, their retention and divergence between the two subgenomes, and their tissue-specific and stress-responsive expression patterns remain unclear. It is also unknown which *BjNDPK* members are responsive to osmotic and salt stress and may therefore represent candidates for subsequent functional investigation.

In this study, we performed a genome-wide identification and characterization of the *BjNDPK* gene family in the *B. juncea* cultivar T84-66. We investigated their chromosomal distribution, phylogenetic relationships, gene structures, conserved motifs, predicted subcellular localization, syntenic relationships, and tissue-specific expression patterns. We further examined the expression responses of *BjNDPK* genes to PEG-induced osmotic stress and salt stress and measured the associated physiological changes in *B. juncea* leaves. In addition, the root-growth responses of wild-type and *atndpk2* mutant *A. thaliana* plants were compared under mannitol-induced osmotic stress as a complementary analysis of a homologous NDPK member. This study establishes a systematic framework for the *BjNDPK* family and identifies candidate genes for future functional studies of abiotic-stress responses in *B. juncea*.

## 2 Materials and methods

### 2.1 Identification of *BjNDPK* family members

To identify *BjNDPK* gene family members, we first downloaded the genome sequences of *B. juncea (*T84-66.V2.0), *Brassica rapa* (CCA.V1.0), and *Brassica nigra* (C2.V1.0) along with their corresponding GFF3 annotation files from the BjuIR database (http://yanglab.hzau.edu.cn/BjuIR) (Zhang et al., 2024). Simultaneously, we obtained protein sequences of reported NDPK genes from *A. thaliana* in the TAIR database (https://www.arabidopsis.org) as references for homology searches (Reiser et al., 2022). Additionally, we downloaded the HMM file (PF00334) for the conserved NDPK domain from the Pfam database (http://pfam.xfam.org) (Mistry et al., 2021). During candidate gene screening, homologous searches were first performed using the BLASTP tool integrated in the BjuIR platform (E-value ≤ 1e−5, identity ≥ 40%, coverage ≥ 70%) against the protein sequences. Subsequently, domain searches were conducted using HMMER 3.0 (http://hmmer.org) based on the PF00334 HMM model (E-value ≤ 1e−5) (Potter et al., 2018). The results from both methods were intersected, and candidate genes underwent manual verification to remove redundant transcripts and sequences lacking conserved domains. This process ultimately identified non-redundant members of the *BjNDPK* gene family.

### 2.2 Chromosomal localization, protein physicochemical properties, and structure prediction of NDPKs

To gain deeper insights into the distribution of *NDPK* genes within the *B. juncea* genome, we extracted the chromosomal location information for each *NDPK* gene from the *B. juncea* GFF3 file provided by BjuIR. This data was then visualized using the “Gene Location Visualize” module within the TBtools-II software (v2.330) (Chen et al., 2023). A distribution map of *BjNDPK* genes was generated based on the chromosomal order of the *B. juncea* T84–66 v2.0 genome. Concurrently, physicochemical properties of 18 NDPK proteins were predicted using the ExPASy ProtParam tool (https://web.expasy.org/protparam/) (Duvaud et al., 2021). Secondary structure analysis and tertiary structure homology modeling of family members were performed using the SOPMA (https://npsa.lyon.inserm.fr/cgi-bin/npsa_automat.pl?page=/NPSA/npsa_sopma.html) and SWISS-MODEL online servers (https://swissmodel.expasy.org) (Waterhouse et al., 2018). Furthermore, the subcellular localization of BjNDPK proteins was predicted using the WoLF PSORT online tool (https://wolfpsort.hgc.jp/) (Horton et al., 2007).

### 2.3 Phylogenetic analysis

To investigate the evolutionary relationships among NDPK proteins, we first performed multiple sequence alignment of the identified BjNDPK protein sequences using the MUSCLE program within MEGA11 software. Based on the alignment results, a phylogenetic tree was constructed using the Neighbor-Joining (NJ) method. Branch confidence was assessed through 1000 bootstrap replicates, with all other parameters set to default values. Finally, the phylogenetic tree was visualized and enhanced using iTOL (https://itol.embl.de/) to more clearly illustrate the evolutionary relationships among *NDPK* gene family members (Letunic and Bork, 2021).

### 2.4 Gene structure, conserved motif, and cis-element analysis

To reveal the conserved motif features and domain distribution of NDPK proteins, we first analyzed the full-length protein sequences of identified *NDPK* gene family members using the online tool MEME (https://meme-suite.org) (Bailey et al., 2015). Subsequently, NCBI CD-Search (https://www.ncbi.nlm.nih.gov/Structure/cdd) was employed for conserved domain identification, confirming the presence of the typical NDPK catalytic domain (Marchler-Bauer et al., 2015). To analyze cis-acting elements in the promoter regions of the *BjNDPK* gene family, we extracted 2000 bp upstream sequences for each gene using TBtools and submitted them to PlantCARE (https://bioinformatics.psb.ugent.be/webtools/plantcare/html/) for element identification (Lescot et al., 2002), focusing on elements associated with stress responses (e.g., drought, cold, wounding), light regulation, and tissue-specific expression. Cis-acting elements in each *BjNDPK* gene promoter were statistically analyzed and categorized. Finally, the gene structures and cis-acting elements of *BjNDPK* genes were visualized using the Gene Structure Visualization plugin in TBtools.

### 2.5 Synteny analysis of *BjNDPK* genes

To investigate the collinearity of *NDPK* genes in the genomes of *B. rapa*, *B. juncea* and *B. nigra*, we performed interspecies protein homology alignment using Diamond (Buchfink et al., 2015), and performed interspecific and intraspecific synteny analyses with MCScanX v1.0.0 (Wang et al., 2012). The intraspecific synteny relationships and genomic tracks were visualized using the ‘Multiple Synteny Plot and Advanced Circos’ functions in TBtools. To assess selective pressures on these genes across species, we calculated Ka/Ks values using Ka/Ks_Calculator 3.0 (Zhang, 2022).

### 2.6 Interactions between microRNAs and *BjNDPK* target genes

To investigate potential relationships between *BjNDPK* genes and microRNAs, we employed the online tool psRNAtarget. This tool was used to predict target relationships between the *BjNDPK* coding sequences and microRNAs from *rapeseed*, *B. nigra*, and *B. rapa* downloaded from the miRBase database (Kozomara et al., 2019). We selected miRNA-target gene combinations with an expectation value ≤3 (Dai et al., 2018). Finally, data visualization was performed using the online tool WeBio (https://www.bioinformatics.com.cn) (Tang et al., 2023).

### 2.7 Enrichment analysis

All annotated genes in the T84-66.V2.0 genome were used as the background set. Gene Ontology (GO) functional annotation and Kyoto Encyclopedia of Genes and Genomes (KEGG) pathway enrichment analyses for the gene family were performed using the clusterProfiler package in R. Statistical significance was calculated based on the hypergeometric distribution test, and differential terms and pathways with *p* < 0.05 were selected.

### 2.8 Tissue expression pattern analysis

To investigate the expression characteristics of *BjNDPK* genes in different tissues of *B. juncea*, we analyzed the expression profiles across 16 tissues (including leaves, seeds, seed coats, callus, roots, stems, pods, pod wall, flower bud, whole seedling, seedling, shoot, nectary, ovule, embryo, and flower) and generated a heatmap of *BjNDPK* tissue expression patterns.

### 2.9 Expression analysis of *BjNDPK* genes under abiotic stress

The *B. juncea* germplasm line ‘23-443-1’, provided by Professor Zhengjie Wan’s research group at the College of Horticulture and Forestry Sciences, Huazhong Agricultural University, Wuhan, China, was used for the controlled stress experiments, including RT-qPCR and physiological assays. At sowing, seeds were pre-germinated in germination trays lined with gauze over Hoagland’s nutrient solution to maintain humidity. Cultivation was conducted under a photoperiod of 16 h light/8 h dark at a constant temperature of 20 °C until day 10. When seedlings reached the two-leaf-one-heart stage, they were subjected to 20% (w/v) PEG 6000 and 200 mM NaCl stress treatments for 12 h. Samples were collected before treatment (CK) and after treatment (12 h), harvesting the leaves. Each treatment included three biological replicates. The obtained samples were used for subsequent RT-qPCR analysis and determination of H_2_O_2_ and CAT content.

Total RNA was extracted from leaves using the Vazyme Total RNA Kit (Nanjing, China). Concentration and purity were assessed using a NanoDrop micro-volume spectrophotometer. First-strand cDNA was synthesized using the TransScript One-Step gDNA Removal and cDNA Synthesis SuperMix Reverse Transcription Kit (Beijing, China). Real-time quantitative PCR (qPCR) was performed on the QuantStudio 5 system using the Vazyme ChamQ Universal SYBR qPCR Master Mix Kit (Nanjing, China). All primers were designed using the Primer 3 online tool (Untergasser et al., 2012). Based on previous studies, *qBjActin*, *Bj18sRNA*, and *BjuTiP41* were selected as candidate reference genes (**Supplementary Table 2**) (Li et al., 2025). Experiments were performed with three biological replicates and three technical replicates. Relative gene expression levels were calculated using the 2^−ΔΔCT^ method.

### 2.10 Analysis of H_2_O_2_ and CAT content in *B. juncea* under abiotic stress

To determine H_2_O_2_ and CAT content in *B. juncea* leaves, enzyme solutions were extracted via homogenization in PBS (w/v = 1 g:9 ml). Absorbance values were measured at 450 nm using the Plant *H_2_O_2_* and CAT ELISA Kit (Jiangsu, China). H_2_O_2_ and CAT contents were calculated from standard curves and expressed as μg/g (fresh weight). Each treatment comprised three biological replicates and three technical replicates.

### 2.11 Root phenotype analysis of the *A. thaliana atndpk2* mutant stress

The *Arabidopsis atndpk2* mutant (SN22241) lines were purchased from Arshare Biotechnology Co., Ltd. (Fujian, China). Wild-type Col-0 and mutant seeds were germinated and grown in parallel on half-strength Murashige and Skoog (1/2 MS) medium under control conditions or on 1/2 MS medium supplemented with 250 mM mannitol to impose osmotic stress. Primary root length was measured 15 days after germination. Both genotypes were included in each treatment, and differences among genotype–treatment combinations were evaluated at *p* < 0.05.

## 3 Results

### 3.1 Identification and chromosomal localization of the *BjNDPK* gene family

**Figure 1:**
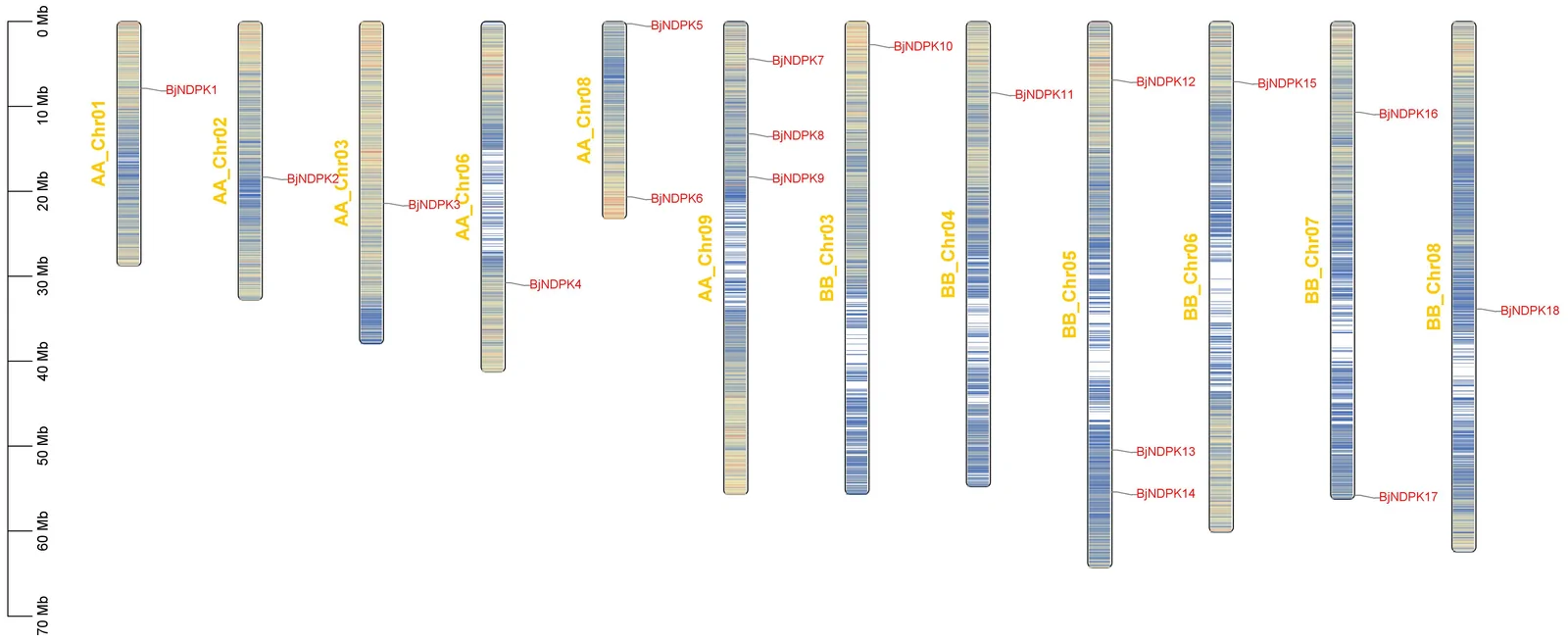
Chromosomal distribution of *BjNDPK* genes in the *B. juncea* genome.

To identify the *NDPK* gene family in *B. juncea*, we screened the *B. juncea* reference genome (T84-66.V2.0) provided by the BjuIR database. This was done based on the reported AtNDPK protein sequences from *A. thaliana*, combined with BLASTP homology alignment and HMMER domain searches. Redundant transcripts and sequences lacking conserved domains were excluded. Ultimately, 18 non-redundant *BjNDPK* genes were identified (**Supplementary Table 1**). Based on chromosome localization information from the genome annotation file, these genes were sequentially named *BjNDPK1* to *BjNDPK18*, and a chromosomal distribution map of *BjNDPK* genes was constructed (**Figure 1**). Results show these 18 *BjNDPK* genes are distributed across 12 chromosomes in the A and B subgenomes. The A subgenome contains 9 genes located on AA_Chr01, AA_Chr02, AA_Chr03 (2 genes), AA_Chr06, AA_Chr08 (2 genes), and AA_Chr09 (3 genes); Subgenome B contained 9 genes located on BB_Chr03, BB_Chr04 (2 genes), BB_Chr05 (3 genes), BB_Chr06, BB_Chr07 (2 genes), and BB_Chr08. Notably, multiple *BjNDPK* genes cluster on certain chromosomes such as AA_Chr09 and BB_Chr05, while no *BjNDPK* members were detected on chromosomes like Chr10.

### 3.2 Phylogenetic analysis of the BjNDPK proteins

**Figure 2:**
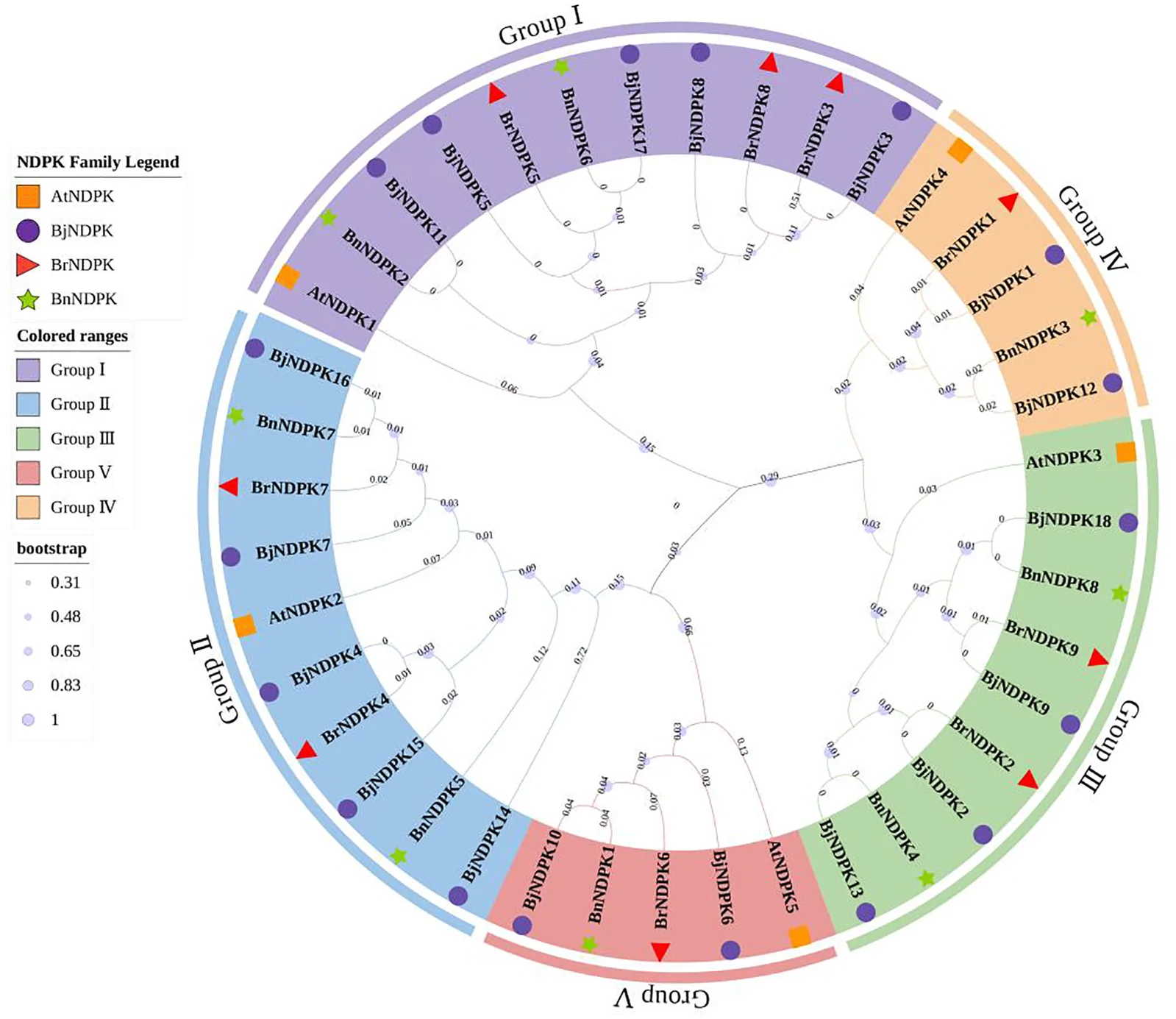
Phylogenetic tree of NDPK proteins from *A. thaliana*, *B. juncea*, *B. nigra* and *B. rapa*.

To investigate the phylogenetic relationships among NDPK proteins across different crops, we classified all NDPK proteins into five groups based on the genetic relationships among 18 BjNDPK proteins, 9 BrNDPK proteins, 8 BnNDPK proteins, and 5 characterized AtNDPK proteins, combined with the position of AtNDPK proteins in the phylogenetic tree: group I, group II, group III, group IV, and group V (**Figure 2**). Five proteins (BjNDPK3, BjNDPK5, BjNDPK8, etc.) clustered in group I. Five proteins (BjNDPK4, BjNDPK14, etc.) clustered in group II. group III contained BjNDPK2, BjNDPK9, BjNDPK13 and BjNDPK18, while the fourth (BjNDPK1 and BjNDPK12) and fifth (BjNDPK6 and BjNDPK10) groups each contain two proteins. Notably, within each branch, most proteins from *B. juncea*, *B. nigra*, and *B. rapa* form tightly clustered subclades, exhibiting extremely close phylogenetic relationships. For example, BjNDPK17 first clusters with BnNDPK6 and then forms a separate branch with BrNDPK5. This suggests that *NDPK* genes have maintained high evolutionary conservation.

### 3.3 Physicochemical properties and subcellular localization prediction of BjNDPK proteins

Protein physicochemical properties revealed that BjNDPK protein amino acid sequences range from 111 to 237 residues, with molecular weights distributed between 12.07 and 25.95 kDa. Theoretical isoelectric points fluctuate between 5.61 and 9.66. Most stability indices are below 40, with the exception of BjNDPK4, which exceeded 40. The aliphatic indices ranged from 76.58 to 91.55, and all grand average of hydropathicity values were below zero, indicating an overall hydrophilic character. Predictions from the WoLF PSORT online tool indicate that group I contains five proteins, all localized to the cytoskeleton. group II comprised five BjNDPK proteins, of which four members were predicted to localize to chloroplasts, whereas the remaining member, BjNDPK14, was predicted to localize to the cytoskeleton. The four proteins in group III and the two proteins in group IV were predicted to localize to mitochondria, whereas the two proteins in group V were predicted to localize to the endoplasmic reticulum (**Supplementary Table 3**). These predictions suggest that BjNDPK proteins may function in different cellular compartments.

### 3.4 Structural prediction of NDPK proteins

**Figure 3:**
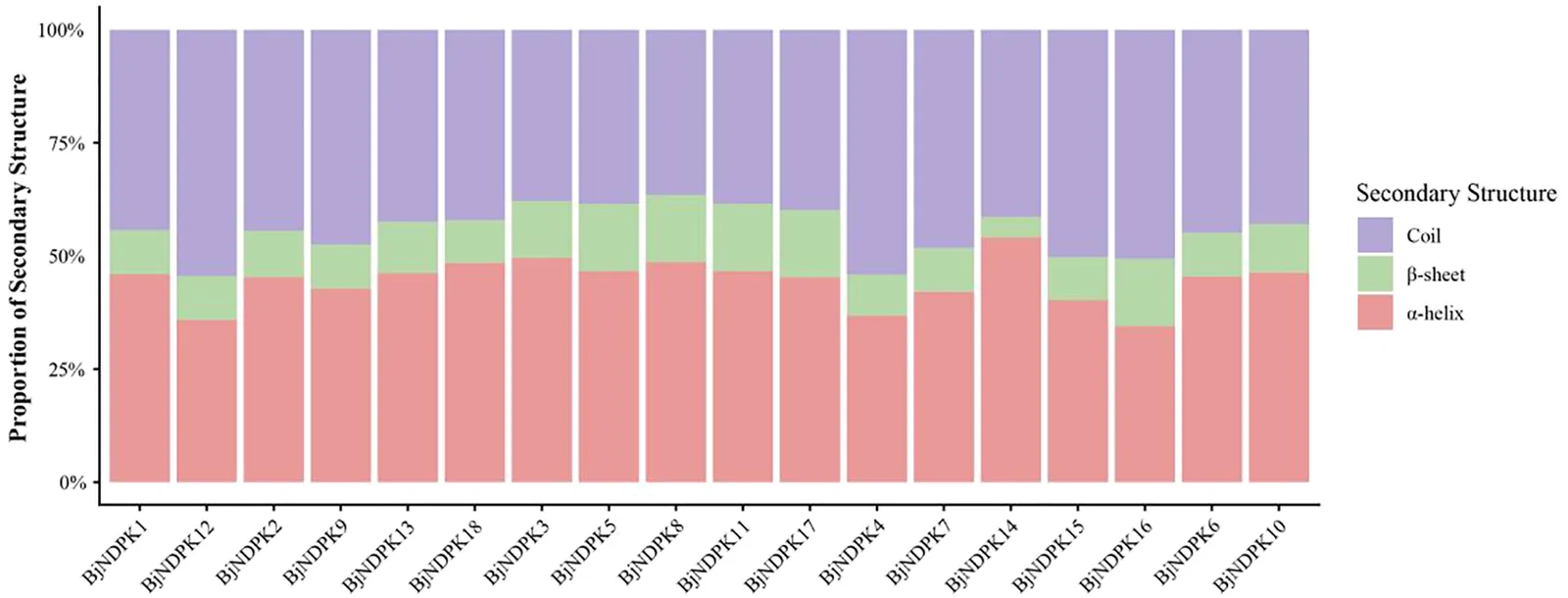
Secondary structure prediction of BjNDPK protein.

Secondary structure analysis revealed (**Figure 3**): α-helix content ranged from 34.47% to 54.08%; β-sheet content ranged from 4.59% to 14.89%; and disordered coils accounted for 37.84% to 54.43%. Among these, BjNDPK14 exhibits the highest α-helix proportion (54.08%), while BjNDPK16 shows the lowest α-helix content (34.47%) and the highest β-sheet proportion (14.89%). Most proteins contained over 40% disordered coils, followed by α-helix, with β-sheet being the least abundant. This distribution may correlate with structural stability relevant to their functions. SWISS-MODEL-based three-dimensional homology modeling revealed that the sequence identities between the predicted models and their respective templates generally exceeded 85% and reached 100% in some cases. Proteins in groups I and IV were predicted to form homohexamers. This is consistent with the crystal structures of their templates (e.g., PDB: 1u8w, 1w7w), which clearly demonstrate the typical hexameric assembly of NDPK proteins: two ring-shaped trimers face each other and interlock to form a flat cylinder with a central channel. In contrast, proteins in groups II, III, and V were predicted to exist in a monomeric configuration (**Supplementary Table 4**; **Supplementary Figure 1**). Despite these variations in oligomeric states among the groups, the overall tertiary structure exhibits significant conservation. This reflects the maintenance of a similar structural framework throughout the evolutionary history of the NDPK family and supports their potential functional homology.

**Figure 4:**
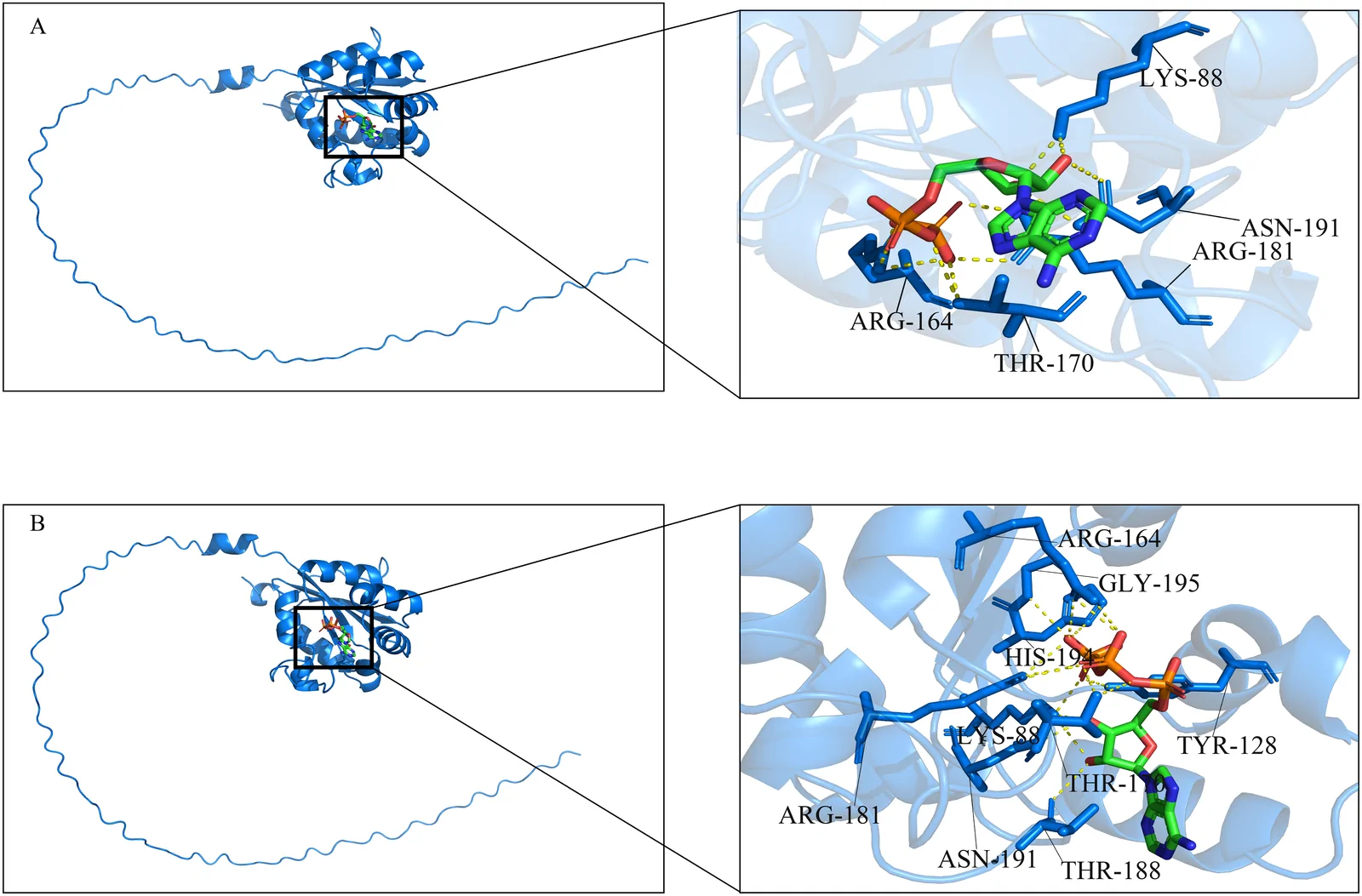
Molecular docking analysis of BjNDPK7 with ADP **(A)** and ATP **(B)**.

Molecular docking analysis revealed distinct binding modes of BjNDPK7 with ATP and ADP. All residue numbers reported below refer to positions in the full-length BjNDPK7 amino acid sequence. ADP is stably anchored primarily through a hydrogen bond network involving residues LYS-88, ARG-164, THR-170, ARG-181, and ASN-191 of BjNDPK7 (**Figure 4A**). In contrast, ATP binding involves a more extensive interaction interface; in addition to the conserved residues ARG-164, THR-170, and ASN-191, it specifically recruits HIS-194, GLY-195, and TYR-128 for ligand recognition (**Figure 4B**). These differences in binding modes suggest that the BjNDPK7 active center maintains basic recognition of the nucleotide phosphate backbone via arginine and threonine residues, while utilizing microenvironmental changes involving histidine and tyrosine residues to distinguish between ATP and ADP, thereby regulating the efficiency of phosphoryl transfer.

### 3.5 Analysis of BjNDPK structure, conserved motifs and promoter cis-acting elements

**Figure 5:**
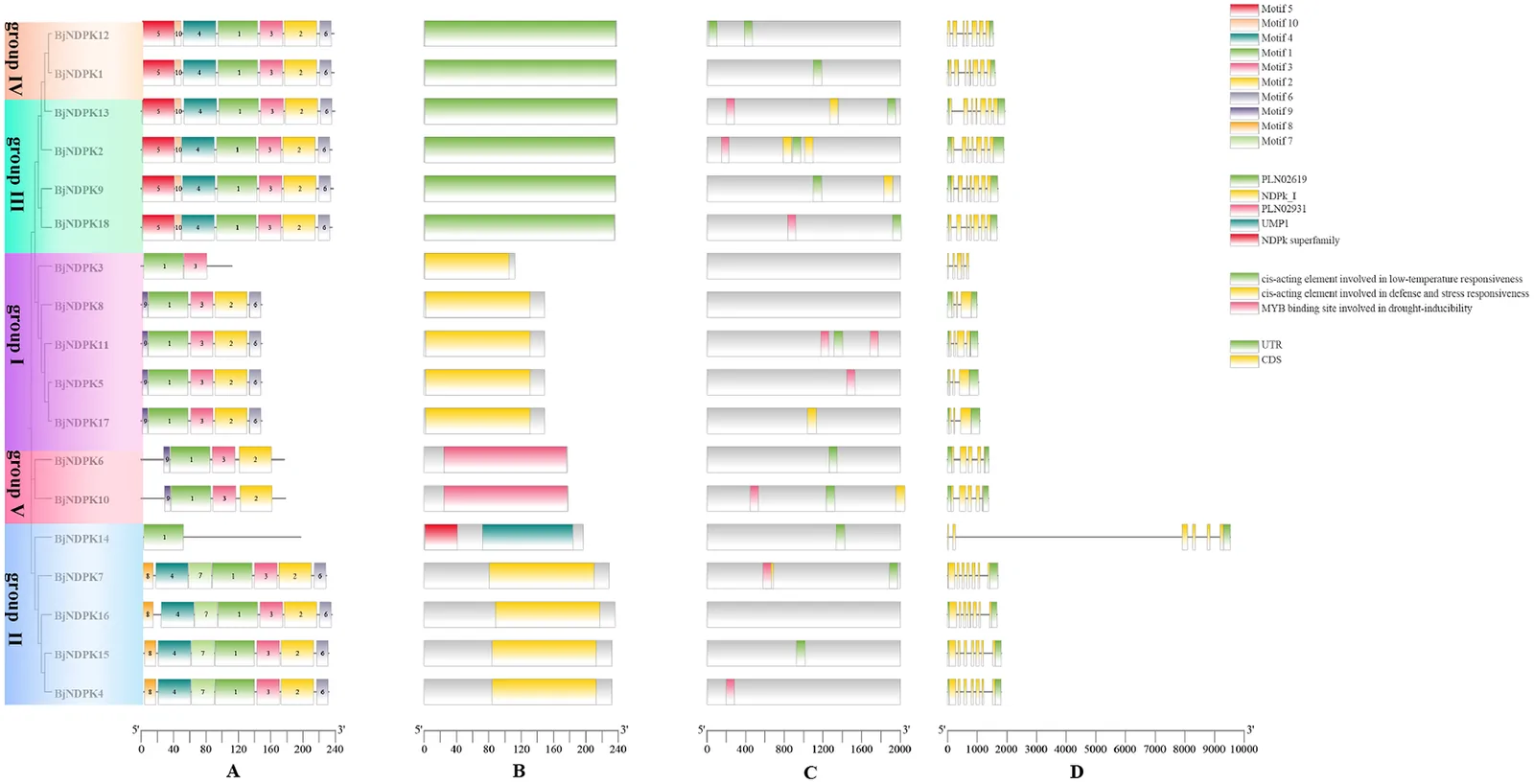
Gene structure analysis of *NDPK* family in *B. juncea*. **(A)** Conserved motifs of BjNDPK family proteins. **(B)** Pfam structure of BjNDPK family proteins. **(C)** Promoter cis-acting element of *BjNDPK* genes. **(D)** The mRNA structure encoded by the *BjNDPK* genes.

The distribution patterns of conserved motifs were analyzed across the protein sequences of 18 *BjNDPK* family members (**Figure 5A**). A total of 10 conserved motifs were identified. Motif 1 was present in all BjNDPK proteins, while motif 3 was found in 17 members, suggesting the critical roles of these two motifs within the *BjNDPK* gene family. Additionally, distinct differences were observed among different branches: motif 9 was specific to groups I and V, motif 8 was specific to group II, motif 5 was specific to group I, motif 10 was specific to group V, and motif 6 was absent only in group V. These group-specific distribution patterns of motifs suggest functional diversification among *BjNDPK* family members during evolution. Analysis of Pfam domain composition (**Figure 5B**) indicates that groups I and III possess the typical NDPK_1 domain, while groups II and IV contain the NDPK-associated PLN02619 domain. Notably, BjNDPK14 possesses an NDPK superfamily domain. In summary, BjNDPK proteins retain highly conserved core components while also showing branch-specific molecular features arising from domain and motif gain or loss.

**Figure 6:**
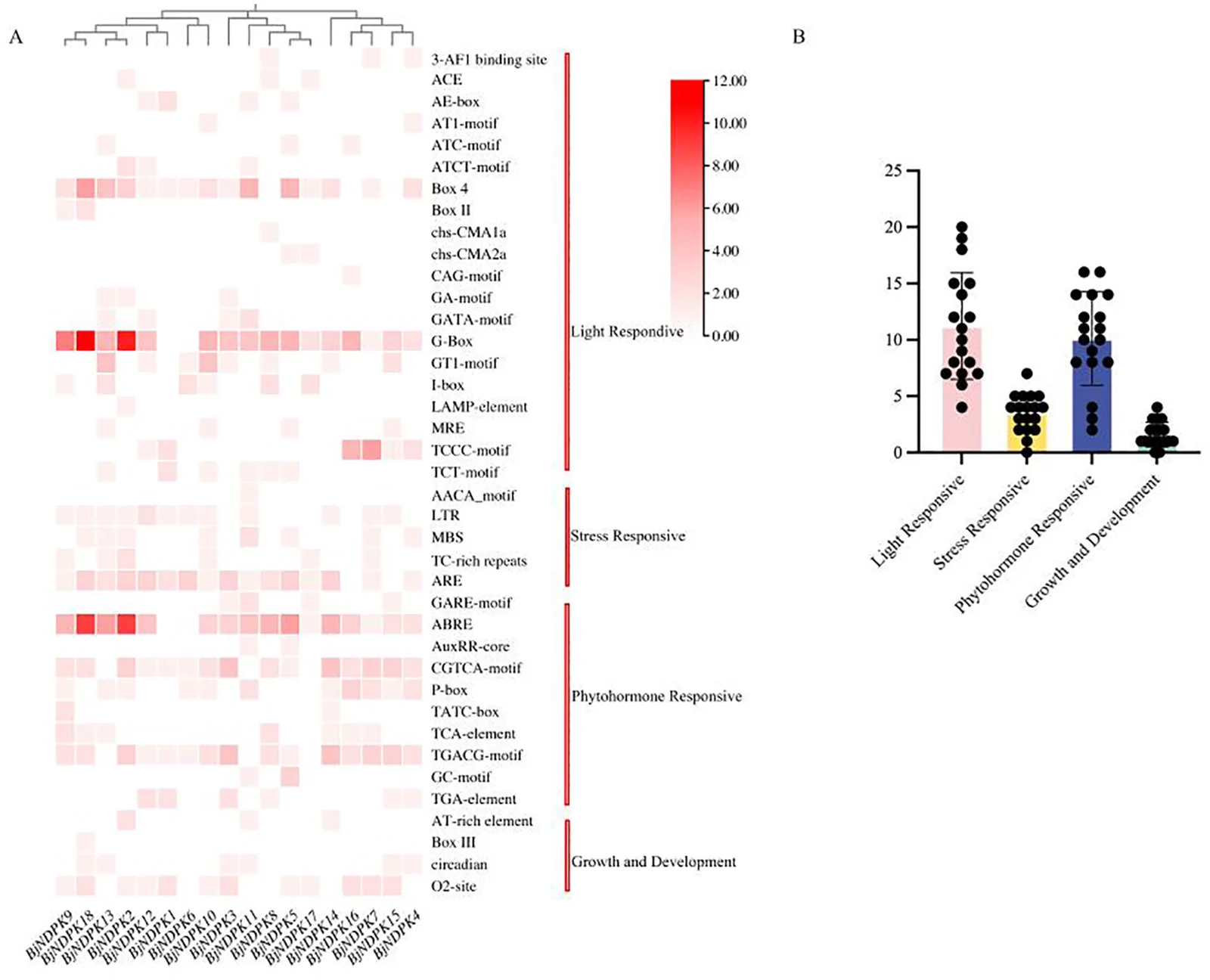
Enrichment and distribution of cis-acting elements in *BjNDPK* promoters. **(A)** Heatmap showing the presence of cis-elements across *BjNDPK* members; **(B)** Quantitative distribution of cis-elements categorized by functional groups.

Intron-exon structure analysis (**Figure 5D**) revealed significant structural differences among members of the *BjNDPK* gene family. All members exhibited a marked 3’ end bias in their CDS. Furthermore, within the same branch, the 3’ CDS length was highly conserved among members, while the 5’ CDS length showed considerable variation, indicating a specific spatial distribution pattern of conservation and variability in the coding region. Promoter cis-acting element analysis revealed that 6 genes (including *BjNDPK2* and *BjNDPK13*) contain cis-acting elements involved in defense and stress responses; 9 genes (such as *BjNDPK2* and *BjNDPK10*) carry drought-induced MYB binding sites, distributed in groups I, II, III, and V; and 12 genes (including *BjNDPK1* and *BjNDPK2*) contain elements related to low-temperature responses, which are present in all phylogenetic groups. These cis-acting elements exhibit specific distribution patterns across different evolutionary branches, suggesting that the *BjNDPK* family may play a potentially functional role in various abiotic stress responses (**Figure 5C**). To systematically compare the compositional characteristics of cis-acting elements in the promoter regions of *BjNDPK* genes and identify element types specifically enriched in particular evolutionary branches, we performed hierarchical cluster analysis on all identified elements and their quantities and constructed a visualization heatmap based on this (**Figure 6A**). The analysis showed that light-responsive elements are widely present in the family. Among them, G-Box elements are distributed in the promoter regions of most genes, especially significantly enriched in the evolutionary branches containing *BjNDPK2*, *BjNDPK13*, *BjNDPK9* and *BjNDPK18*. In contrast, *BjNDPK1* and *6* not only lack G-Box elements but also have extremely low overall light-responsive element content. Among stress-related elements, anaerobic response elements (AREs) dominate the distribution. Furthermore, the abscisic acid response element (ABRE), associated with plant hormones, is ubiquitous throughout the family. Interestingly, ABRE and the light-responsive element G-box show a high degree of similarity in their distribution across all *BjNDPK* gene promoters, suggesting that they may synergistically play a role in the cross-regulation of light and ABA signaling pathways. We also identified several cis-regulatory elements related to plant growth and development, including AT-rich elements and Box III associated with chromatin structure and organization, circadian elements regulating biological rhythms, and O_2_-sites specifically regulating the expression of seed storage protein genes. Analysis of the four types of elements revealed that light-responsive elements were the most numerous, followed by hormone-responsive elements, whereas growth- and development-related elements were the least abundant (**Figure 6B**).

### 3.6 Synteny analysis of the *BjNDPK* gene family

**Figure 7:**
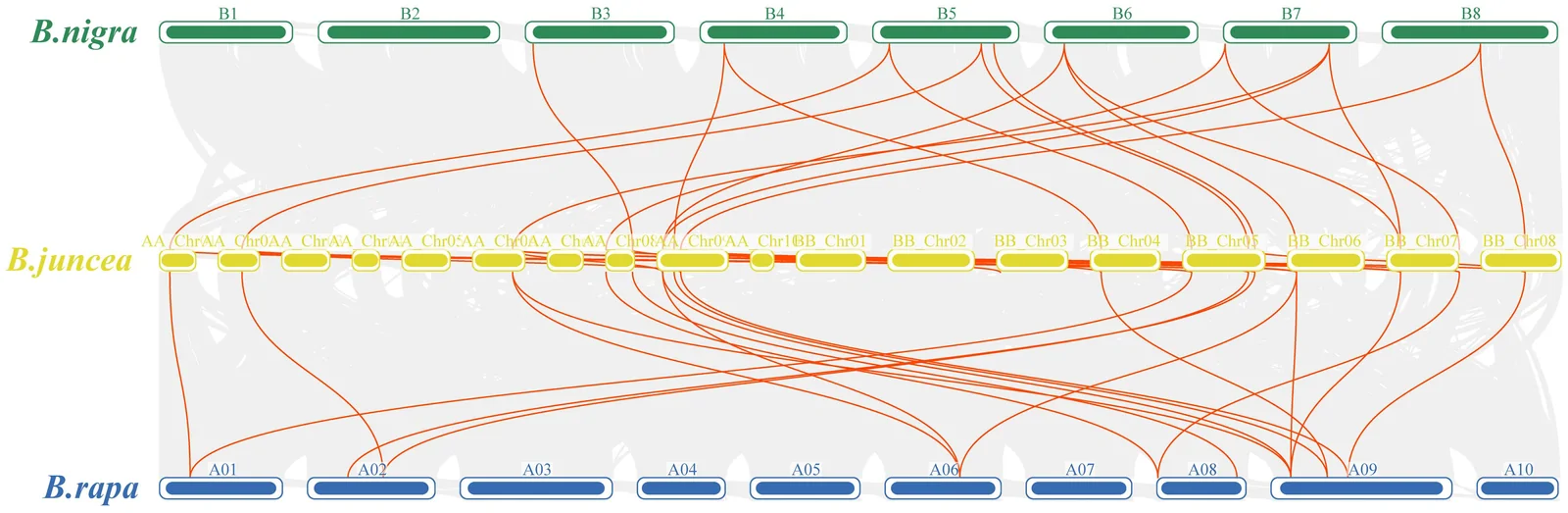
Collinearity of *NDPK* genes in *B. juncea* (yellow), *B. nigra (*green) and *B. rapa* (blue).

**Figure 8:**
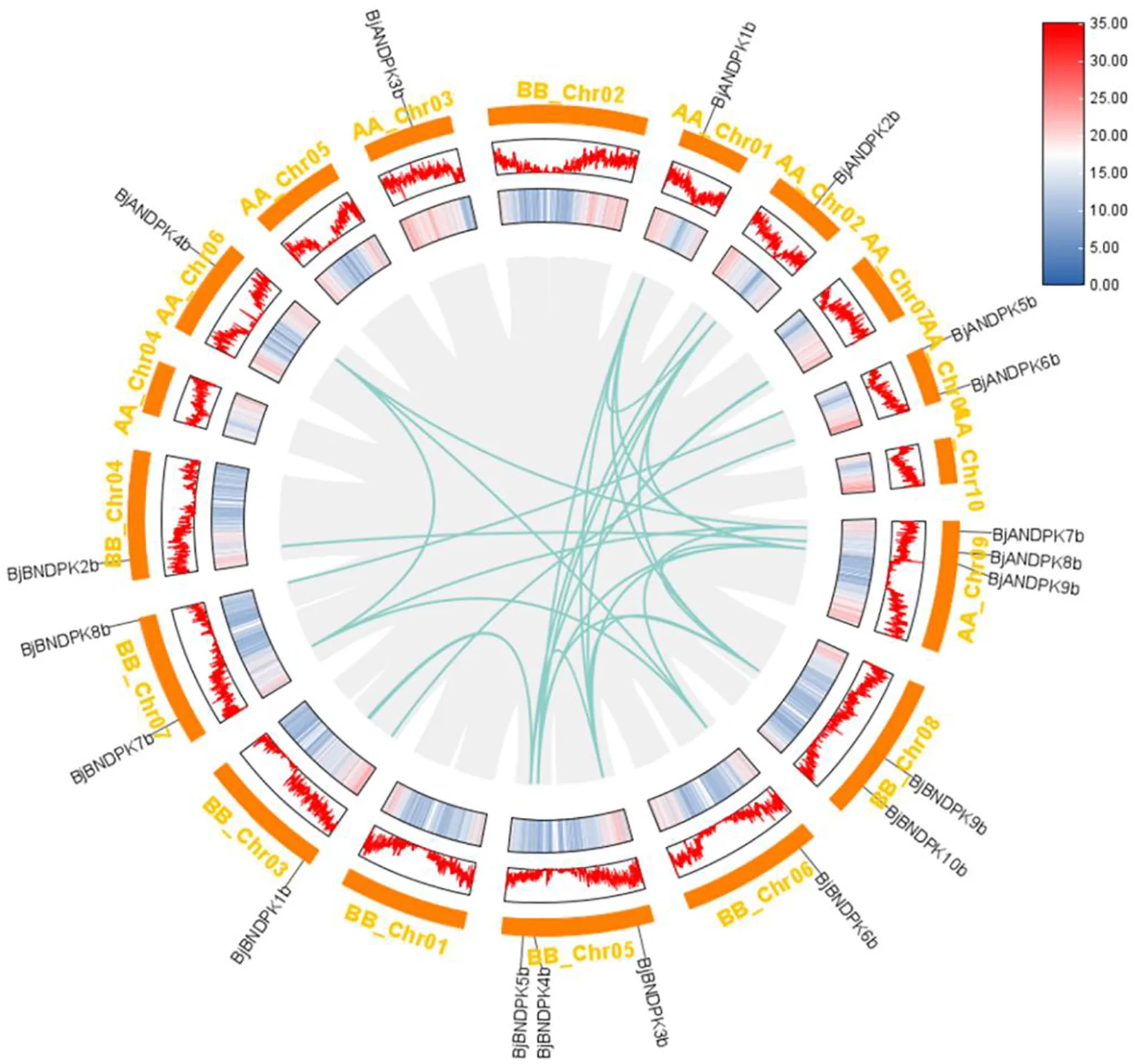
Collinearity of *BjNDPK* genes. The circles in the figure from inside to outside represent the gene density, GC ratio and chromosome length of the *B. juncea* genome.

To further investigate the expansion mechanism of the *BjNDPK* gene family in *B. juncea* and its evolutionary homology with the genomes of *B. rapa* and *B. nigra*, we conducted a systematic collinearity analysis using MCScanX software. The interspecific collinearity analysis (**Figure 7**) reveals 18 pairs of *NDPK* homologous genes between *B. nigra* and *B. juncea*; and 19 pairs of *NDPK* homologous genes between *B. rapa* and *B. juncea*. Notably, the *BjNDPK* genes in the A subgenome of *B. juncea* showed collinearity not only with the *B. rapa* (AA) genome but also with the *B. nigra (*BB) genome. Genes in the B subgenome also showed similar collinearity. This supports the hypothesis that *B. juncea* originated as an allotetraploid (AABB) formed by heteropolyploidization of *B. rapa* and *B. nigra and* also demonstrates the high conservation of the *NDPK* family. Intraspecific collinearity analysis identified 24 collinear gene pairs (**Figure 8**). These collinear relationships not only existed within the same subgenome but were also widely distributed between subgenomes, indicating that the *BjNDPK* family underwent complex inter-subgenome rearrangement and retention after the whole-genome duplication (WGD) event in *B. juncea*. Notably, no collinearity was detected in *BjNDPK3*, possibly due to recent tandem duplication or transposition events causing it to deviate from its ancestral collinearity region. To further investigate the evolutionary selection pressure on *BjNDPK* collinear gene pairs, we calculated the ratio of nonsynonymous substitution rate (Ka) to synonymous substitution rate (Ks) (Ka/Ks). Analysis of the 29 collinear gene pairs involved showed that the Ka/Ks values were significantly less than 1 (0.027-0.246, **Supplementary Table 5**), indicating that this gene family has undergone strong purifying selection during evolution.

### 3.7 miRNA–*BjNDPK* regulatory network

**Figure 9:**
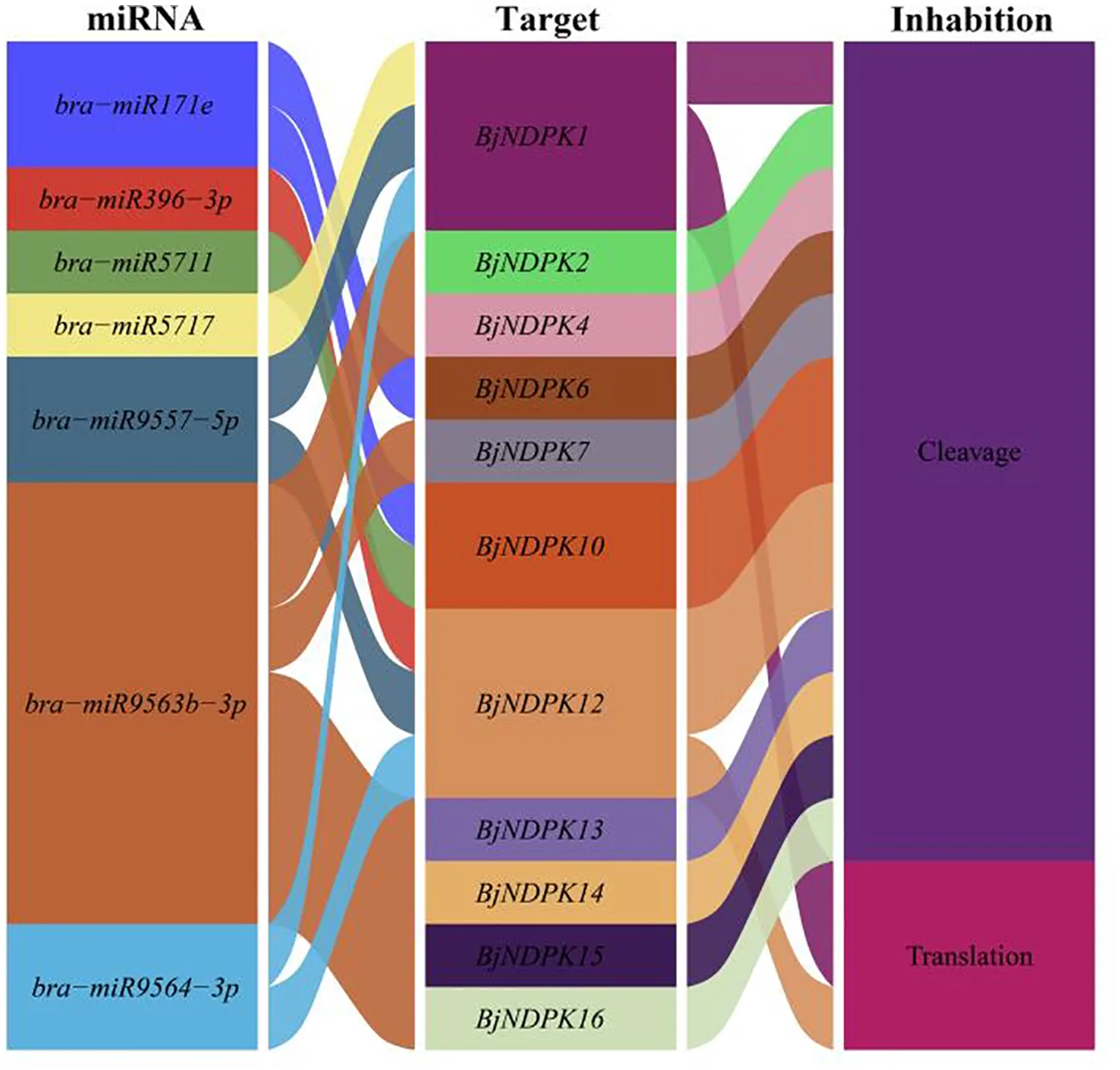
Analysis of the interaction between microRNA and *BjNDPK* genes.

To investigate the post-transcriptional regulatory mechanism of *BjNDPK* genes, we performed a predictive analysis of miRNA targeting relationships among *BjNDPK* family members based on the miRNA sequences of its close relative (*B. rapa*) and visualized the complex interactions using impact maps. A total of 7 different miRNAs were identified that can target and regulate 10 *BjNDPK* genes, forming 16 specific regulatory pairs. The results showed that there are two main interaction relationships between miRNAs and genes, and two regulatory modes: ‘one-to-many’ and ‘many-to-one’. Specifically, bra-miR9563b-3p can regulate up to 7 target genes, while *BjNDPK1* and *BjNDPK12* were predicted to be targeted by three miRNAs (**Figure 9**). This suggests that *BjNDPK* genes may be finely regulated at the post-transcriptional level.

### 3.8 GO and KEGG enrichment analyses of the *BjNDPK* genes

**Figure 10:**
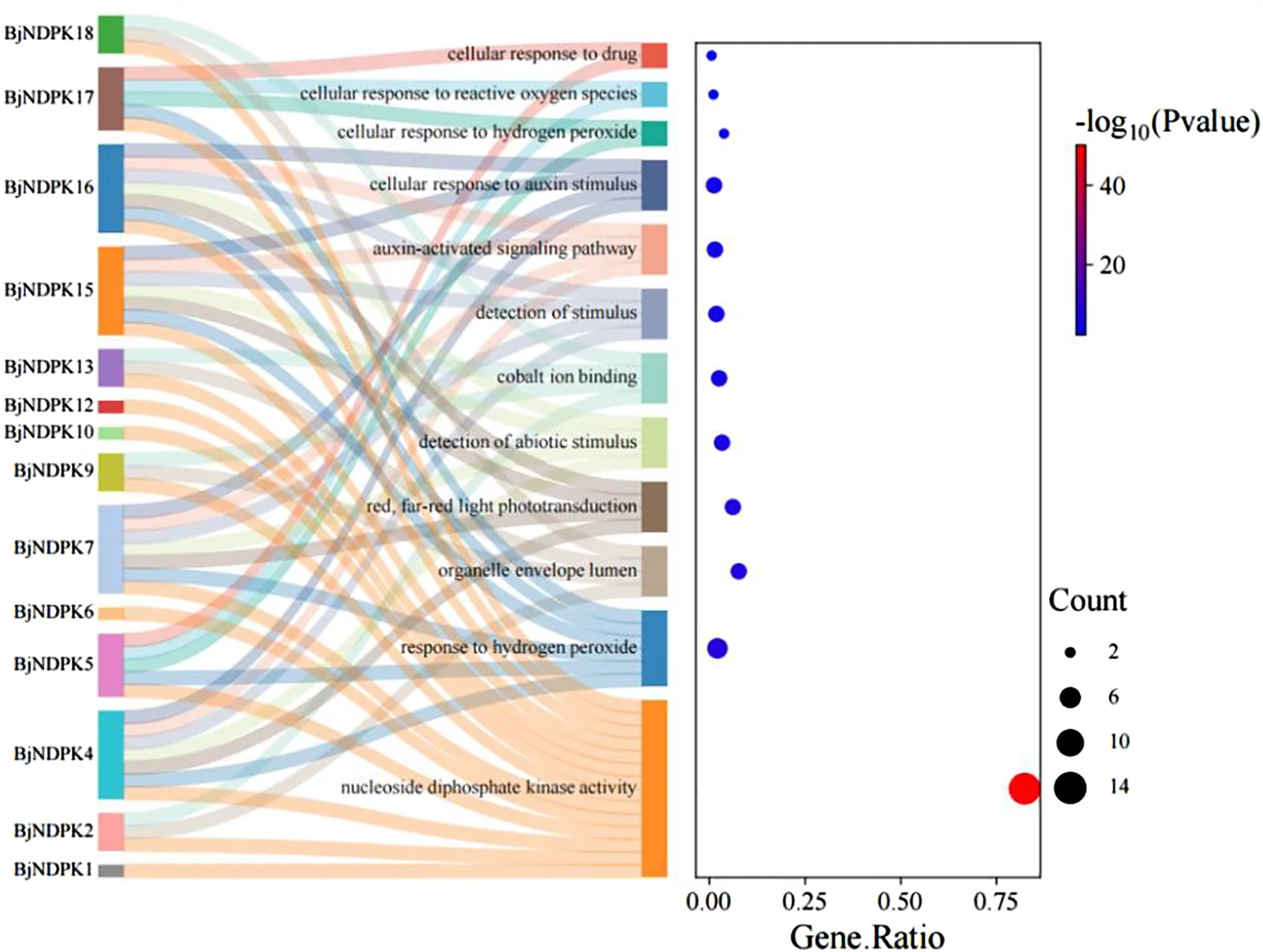
GO functional annotation and enrichment analysis of *BjNDPK* genes.

To elucidate the potential biological functions of the *BjNDPK* gene family in *B. juncea*, systematic Gene Ontology (GO) and Kyoto Encyclopedia of Genes and Genomes (KEGG) enrichment analyses were performed. The results indicated that the majority of *BjNDPK* members possess significant nucleoside diphosphate kinase activity and are involved in diverse biological processes. Furthermore, the implication of several members in the auxin signaling pathway and cobalt ion binding may be associated with oxidative stress responses, light signal perception, and hormonal regulation. Notably, group III genes were specifically enriched in the “organelle envelope lumen”. Given the semi-autonomous nature of chloroplasts, this localization implies their potential involvement in photosynthesis-related energy metabolism or metabolite transport within the chloroplast intermembrane space. Conversely, group V genes were significantly clustered in “cellular response to drug” and reactive oxygen species (ROS) metabolism-related terms. Although their precise biological functions remain to be elucidated, this unique enrichment pattern suggests a possible association with responses to xenobiotic stress or oxidative stress triggered by secondary metabolite accumulation, thereby providing valuable clues for future functional characterization (**Figure 10**). Furthermore, KEGG pathway analysis revealed that, with the exception of a few genes, group I, group II and group III were significantly enriched in core networks, including pyrimidine metabolism, MAPK signaling pathway, purine metabolism, and membrane transport (**Supplementary Table 6**). Collectively, these findings suggest that the *BjNDPK* gene family may synergistically respond to complex environmental stimuli by regulating nucleotide metabolic homeostasis and mediating signal cascades.

### 3.9 Tissue-specific expression pattern analysis

**Figure 11:**
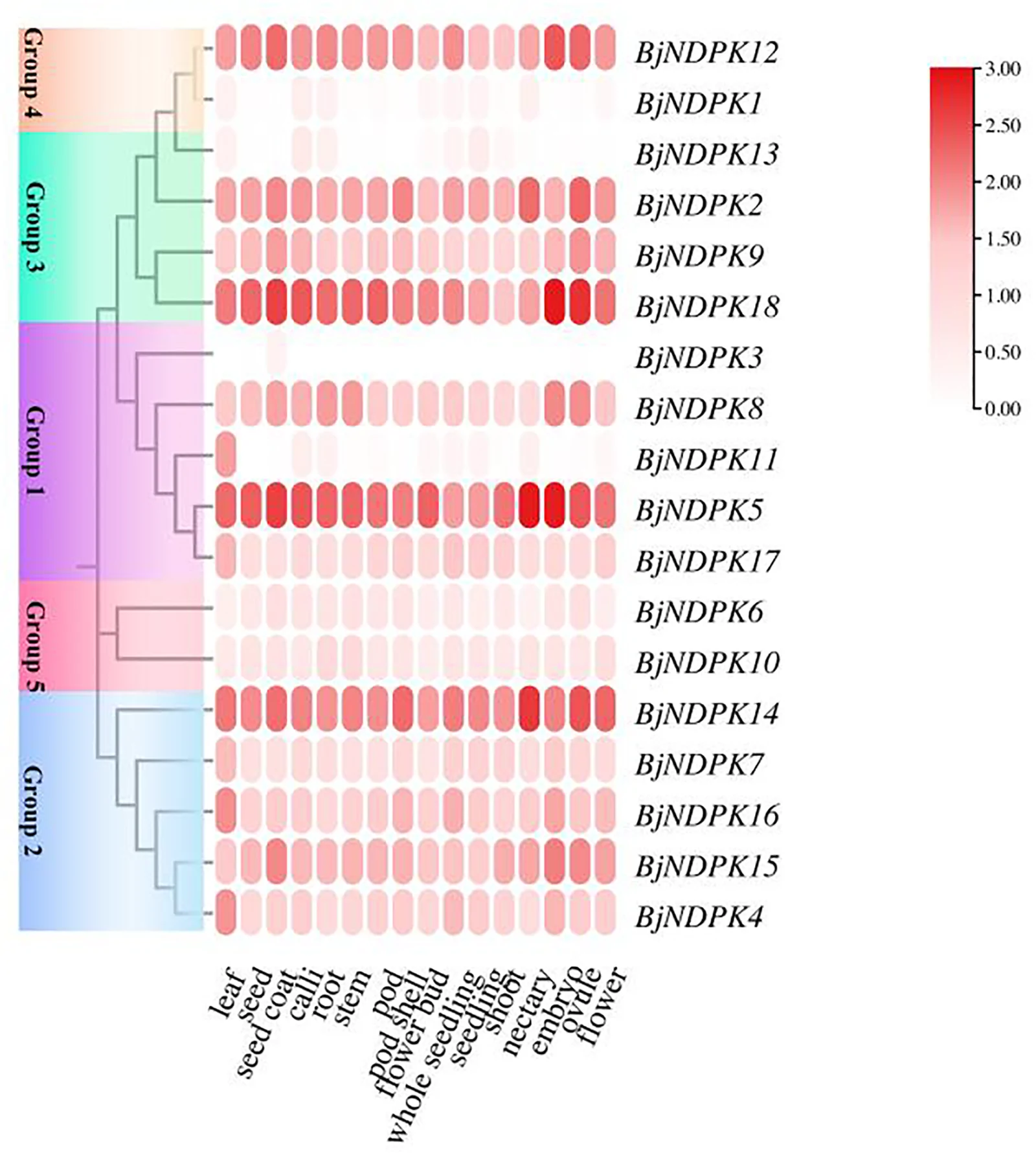
Tissue-specific expression profiles of *BjNDPK* genes across 16 tissues: leaf, seed, seed coat, calli, root, stem, pod, pod shell, flower bud, whole seedling, seedling, shoot, nectary, embryo, ovule and flower.

To investigate the expression patterns of *BjNDPK* genes in different tissues of the *B. juncea* variety T84-66, we integrated transcriptome data (expressed as TPM values) from 16 tissue types of this variety and constructed a gene expression heatmap after normalization (log_10_ (TPM + 1)) (**Figure 11**). These data indicate that members of the *BjNDPK* family were expressed in multiple tissues, exhibiting certain tissue specificity and functional differentiation. Specifically, *BjNDPK1*, *BjNDPK13*, *BjNDPK3*, and *BjNDPK11* showed extremely low or undetectable expression levels in most tissues, suggesting they may have specific regulatory mechanisms or limited function. *BjNDPK17*, *BjNDPK6*, *BjNDPK10*, and *BjNDPK7* showed relatively uniform expression across tissues but low overall abundance. In contrast, several genes, including *BjNDPK12*, *BjNDPK2*, and *BjNDPK18*, showed relatively high expression levels across most tissues, particularly in the seed coat, embryo, and ovule, suggesting their potential involvement in seed development and reproduction.

### 3.10 Expression analysis of the *BjNDPK* gene family in response to abiotic stress based on stable internal references

**Figure 12:**
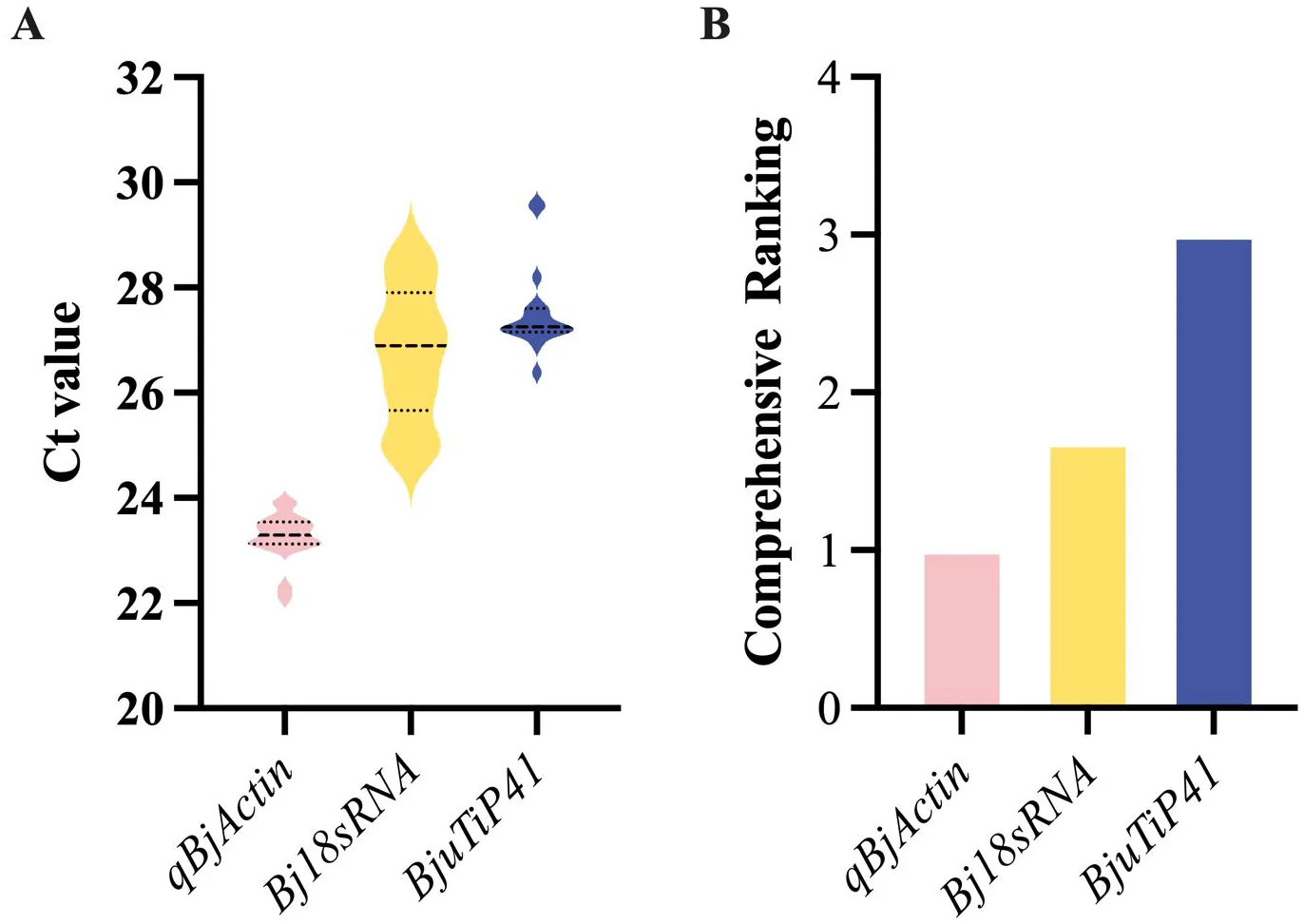
Evaluation of reference gene stability in *B. juncea* based on Ct values and comprehensive stability scores. **(A)** Distribution of Ct values for candidate reference genes; **(B)** Comprehensive stability scores calculated using multiple algorithms.

To investigate the expression of the *BjNDPK* gene family under abiotic stress, we used RT-qPCR to analyze the expression changes of each gene in *B. juncea* seedlings under drought stress. To ensure the accuracy of the qPCR data, we evaluated the stability of three selected candidate internal control genes (*qBjActin*, *Bj18sRNA*, and *BjuTiP41*) using geNorm, NormFinder, and BestKeeper software. The internal control stability matrix showed that *qBjActin* exhibited the most stable expression under different stress treatments and was ultimately selected for subsequent standardized calculations of target gene expression levels (**Figure 12**; **Supplementary Table 7**).

**Figure 13:**
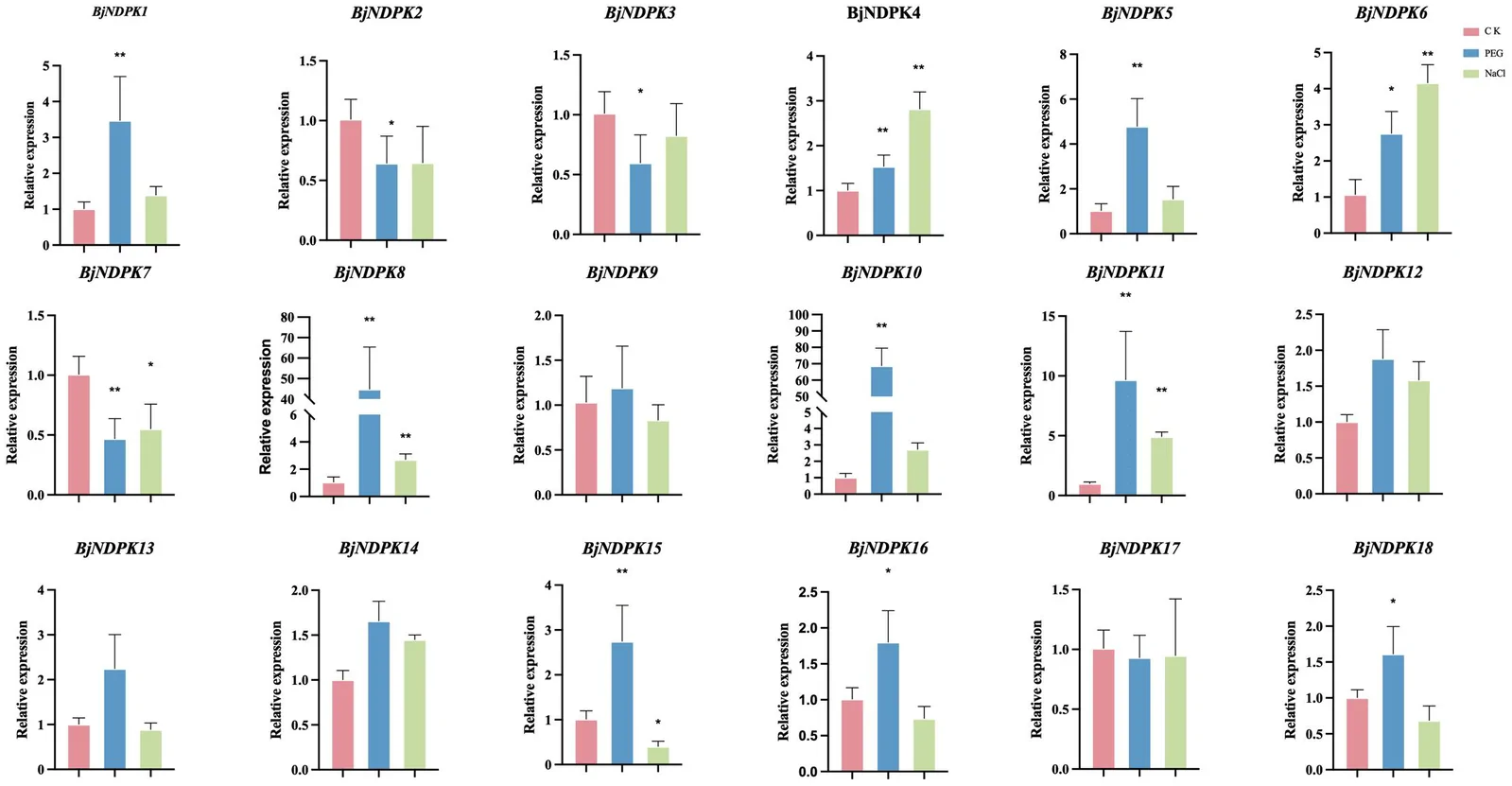
The relative expression of *BjNDPK* genes in 20% (w/v) PEG 6000 and 200 mM NaCl after 0 and 12 h. Data represent the mean ± standard error for three biological experiments. Statistical significance was determined using Student’s t-test. *significant differences between treatments at *p* ≤ 0.05. **significant differences between treatments at *p* ≤ 0.01.

The expression levels of the 18 *BjNDPK* family members under drought and salt stress were analyzed (**Figure 13**). The results showed that under drought stress, the relative expression levels of 8 genes, including *BjNDPK1*, *4*, *5*, *8*, *10*, and *11*, were significantly upregulated (*p* < 0.01), and those of *BjNDPK6*, *16*, and *18* were significantly increased. In contrast, the expression levels of *BjNDPK2*, *3*, and *7* were significantly downregulated (*p* < 0.05). Under salt stress conditions, the relative expression levels of *BjNDPK4*, *6*, and *11* were significantly upregulated (*p* < 0.05), whereas those of *BjNDPK7* and *8* were significantly downregulated (*p* < 0.05). At 12 h after treatment, *BjNDPK* genes exhibited distinct expression patterns under drought stress and salt stress. However, because only a single post-treatment time point was examined, the temporal dynamics of these responses could not be determined.

### 3.11 Analysis of changes in hydrogen peroxide and CAT content under abiotic stress

**Figure 14:**
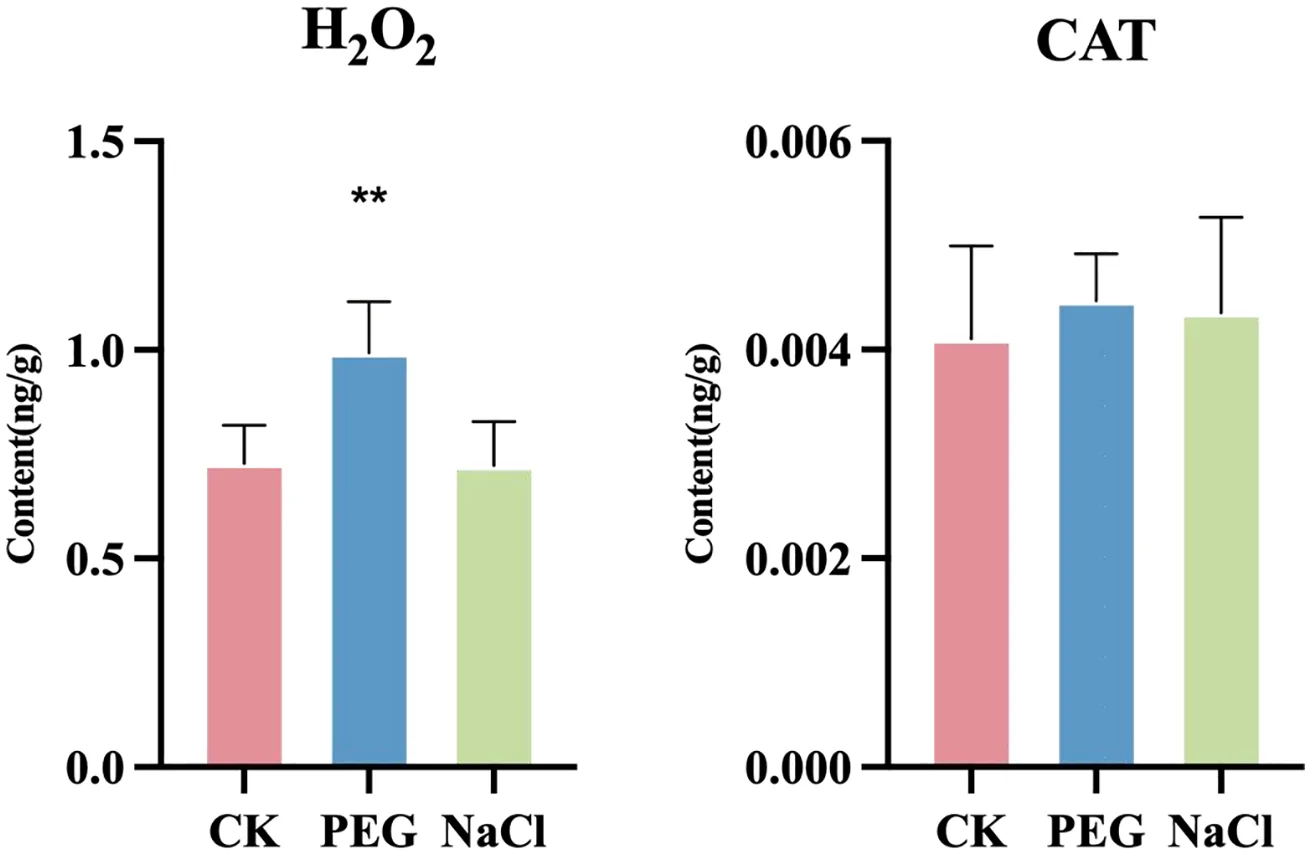
Changes in H_2_O_2_ and CAT content in *B. juncea* leaves under CK, 20% PEG 6000 and 200 mM NaCl stress.

Standard curves for H_2_O_2_ and CAT were first established, yielding correlation coefficients greater than 0.99 for both. Compared to the control, H_2_O_2_ content increased significantly under drought stress, whereas no significant change was observed under salt stress. Moreover, CAT content showed no significant differences under either stress condition (**Figure 14**).

### 3.12 Root phenotype analysis of the *A. thaliana atndpk2* mutant

**Figure 15:**
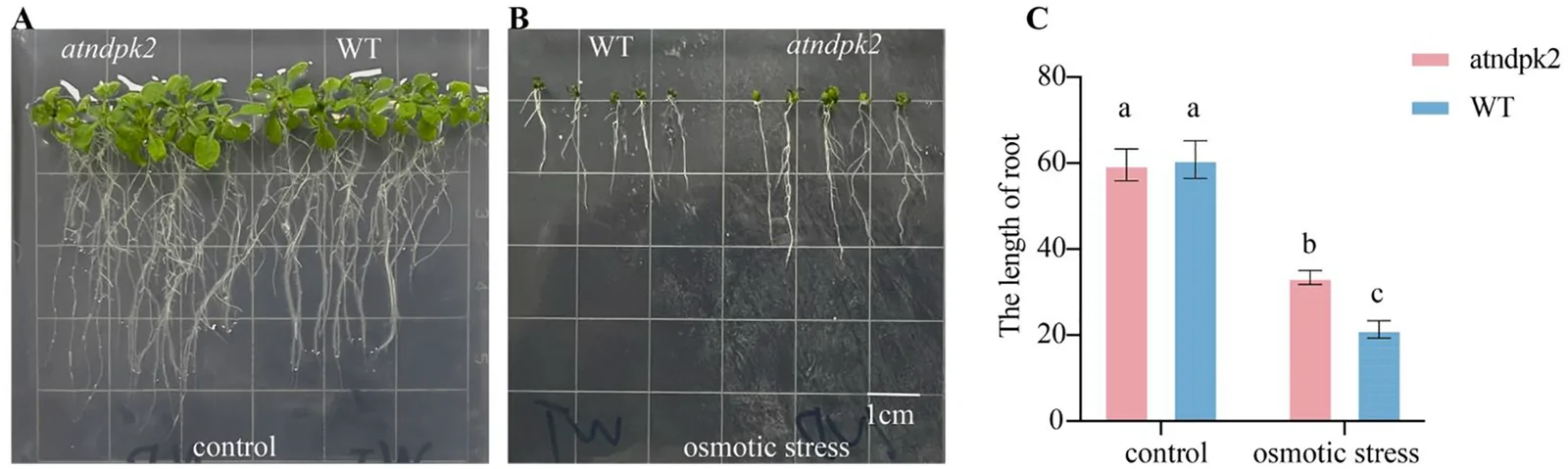
Phenotypic analysis of the *atndpk2* mutant in *A. thaliana*. **(A)** Phenotype of the *atndpk2* mutant in 1/2 MS (control), **(B)** Phenotype of the *atndpk2* mutant in 1/2 MS + 250 mM mannitol (osmotic stress), **(C)** Root length of the *atndpk2* mutant at 15 days old. Statistical differences between each group were determined using LSD. The letters above the columns indicate significant differences (*p* < 0.05).

WoLF PSORT online database predictions indicate that the AtNDPK2 protein is localized in the chloroplast. To investigate its role in root development and drought stress responses, the purchased *atndpk2* mutant was used. Phylogenetic analysis revealed that AtNDPK2 is the closest ortholog to BjNDPK7 in *B. juncea*. We evaluated the root length phenotypes of both the mutant and wild-type (WT) plants under normal conditions (1/2 MS medium) and simulated drought stress (1/2 MS supplemented with 250 mM mannitol) (**Figures 15A, B**). Under normal growth conditions, no significant difference in root length was observed between the mutant (58.64 ± 3.21 mm) and WT (60.93 ± 3.58 mm) plants. However, under drought stress, the root length of WT plants was significantly reduced to 22.07 ± 1.85 mm, whereas the mutant maintained a significantly longer root length of 34.08 ± 2.13 mm (**Figure 15C**).

## 4 Discussion

*B. juncea* (2n = 36, AABB) is an allotetraploid species derived from the hybridization and genome doubling of *B. rapa* (AA) and *B. nigra* (BB) (Kang et al., 2021). In this study, 18 *BjNDPK* genes were identified, with comparable numbers distributed between the A and B subgenomes. The interspecific and intraspecific syntenic relationships suggest that many *BjNDPK* genes were inherited from the diploid progenitors and retained following polyploidization. Moreover, the Ka/Ks ratios of the collinear gene pairs were lower than 1, indicating that these genes have mainly been subjected to purifying selection. This result is consistent with the conservation of the core phosphotransfer function of NDPK proteins (Kang et al., 2021; Feng et al., 2024). Nevertheless, their uneven chromosomal distribution and group-specific differences in gene structure and conserved motifs suggest that individual family members may have undergone regulatory or functional divergence after duplication. These patterns provide evidence of evolutionary conservation and diversification but do not, by themselves, demonstrate sub functionalization.

The structural and subcellular localization analyses further support both conserved and potentially divergent features within the BjNDPK family. Most BjNDPK proteins contained the conserved NDPK catalytic domain and showed high predicted structural similarity to known NDPK proteins, supporting their potential capacity for nucleotide phosphate transfer (Morgan et al., 2020). The presence of group-specific domains and motifs suggests that certain isoforms have evolved specialized functions beyond energy metabolism, such as redox regulation, protein–protein interaction, and long-distance signal transduction. The diverse subcellular localization patterns further imply that BjNDPK proteins participate in organelle crosstalk critical for stress signaling (Dorion and Rivoal, 2015). However, these predicted localizations and structures require experimental verification.

Expression profiling revealed divergent responses of *BjNDPK* genes to drought and salt treatment: most genes were induced, whereas a small subset was repressed. Because the RT-qPCR analysis was restricted to aboveground tissues, the present results do not reveal the root-specific expression responses of *BjNDPK* genes. Given that roots are major sites of osmotic and salinity stress perception, future studies should analyze root and shoot tissues separately at multiple time points. The content of H_2_O_2_ in leaf increased under PEG treatment, whereas the content of CAT did not change significantly. These associations are consistent with, but do not prove, roles for individual *BjNDPK* genes in stress- or ROS-related responses. Furthermore, the genome-wide analyses were based on the T84–66 reference genome, whereas RT-qPCR and physiological assays were performed using the germplasm line 23-443-1. Genotypic differences between these materials may contribute to variation in stress-responsive expression.

Promoter analysis identified multiple cis-acting elements associated with ABA, drought, light, low-temperature, and other environmental responses. The presence of these elements was broadly consistent with the stress-responsive expression observed in the RT-qPCR assays. Several *BjNDPK* genes were induced by PEG or NaCl treatment, whereas other members were repressed, indicating that the family does not respond uniformly to abiotic stress. The different expression directions among family members may reflect divergence in their upstream regulatory mechanisms following gene duplication. In particular, genes that responded to both PEG and NaCl treatment may represent candidates involved in shared osmotic-stress responses, whereas treatment-specific responses may be associated with pathways activated preferentially by osmotic stress or ionic stress. Moreover, promoter cis-element analysis revealed a striking co-occurrence of G-box and ABRE motifs, which may be associated with light signaling and ABA-mediated drought responses. The co-occurrence of these predicted cis-elements suggests that *BjNDPK* genes may be coordinately regulated by light- and ABA-responsive pathways under osmotic and salt stress (Hayashi and Kinoshita, 2011). Nevertheless, the presence of predicted promoter elements cannot establish direct transcriptional regulation. Promoter-reporter assays and transcription-factor-binding experiments will be needed to determine whether these cis-elements mediate the observed expression responses.

Comparative phenotypic analysis was performed using the *A. thaliana atndpk2* mutant. Under osmotic stress, the mutant maintained significantly greater primary root elongation than wild-type Col-0, whereas no significant difference was observed under control conditions. These results suggest that AtNDPK2 is associated with the regulation of root growth under osmotic stress. Phylogenetic analysis identified BjNDPK7 as the closest *B. juncea* ortholog of AtNDPK2, and *BjNDPK7* was downregulated in leaves following PEG treatment. The phenotypic response of the *atndpk2* mutant and the stress-responsive expression of *BjNDPK7* are therefore consistent with the possibility of a related role in osmotic-stress responses. However, these observations do not establish functional conservation or a shared negative regulatory mechanism. Because root tissues were not included in the RT-qPCR analysis, the present data also cannot determine whether *BjNDPK7* participates in root-specific stress perception or root-to-shoot signaling. Direct genetic validation and root-specific expression analysis in *B. juncea* are required to test this hypothesis (Ma et al., 2022).

Overall, the integration of evolutionary, structural, promoter, and expression analyses identifies several *BjNDPK* genes as candidates associated with osmotic- and salt-stress responses. The results establish a framework for prioritizing individual family members for subsequent functional analysis rather than demonstrating a conserved regulatory mechanism for the entire *BjNDPK* family.

## 5 Conclusions

In this study, we performed a comprehensive genome-wide identification and characterization of the *BjNDPK* gene family in *B. juncea*, identifying 18 genes classified into five phylogenetic groups. Structural and evolutionary analyses indicated that the family expanded primarily through segmental duplication and underwent strong purifying selection, preserving core catalytic functions while allowing for structural divergence in oligomeric states and subcellular localization. Crucially, integrated analyses revealed predicted associations of *BjNDPK* genes with stress-responsive cis-elements and miRNAs, suggesting their potential involvement in plant development and abiotic-stress responses. Furthermore, the *A. thaliana atndpk2* mutant maintained greater root elongation than wild-type Col-0 under mannitol-induced osmotic stress. Although the heterologous mutant phenotype does not directly establish the functions of individual *BjNDPK* genes, these findings provide a systematic foundation and candidate genes for future functional studies of abiotic-stress responses in *B. juncea*.

## Data Availability

The original contributions presented in the study are included in the article/**Supplementary Material**. Further inquiries can be directed to the corresponding authors.

## Supplementary material

Supplementary Table 1 Sequence information of *BjNDPK* genes.

Supplementary Table 2 Primer sequences used for qPCR analysis of *BjNDPK* gene family members and reference genes.

Supplementary Table 3 Physicochemical properties and subcellular localization prediction of BjNDPK proteins.

Supplementary Table 4 Tertiary structure modeling information of NDPK protein.

Supplementary Table 5 Ka/Ks value of *B. juncea*.

Supplementary Table 6 KEGG Functional Annotation and Enrichment Analysis of *BjNDPK* genes.

Supplementary Table 7 The stability of 3 different candidate reference genes.

Supplementary Figure 1 Prediction of the tertiary structure of the NDPK protein.
