# Supplementary material for: Genome-wide identification and expression divergence of the NDPK gene family in Brassica juncea under abiotic stress
Source: Front Plant Sci. 2026 Aug 6;17:1917522. doi: 10.3389/fpls.2026.1917522 (PMC13490171; doi:10.3389/fpls.2026.1917522)
Supplement: Supplementary file 1 [file DataSheet1.pdf]

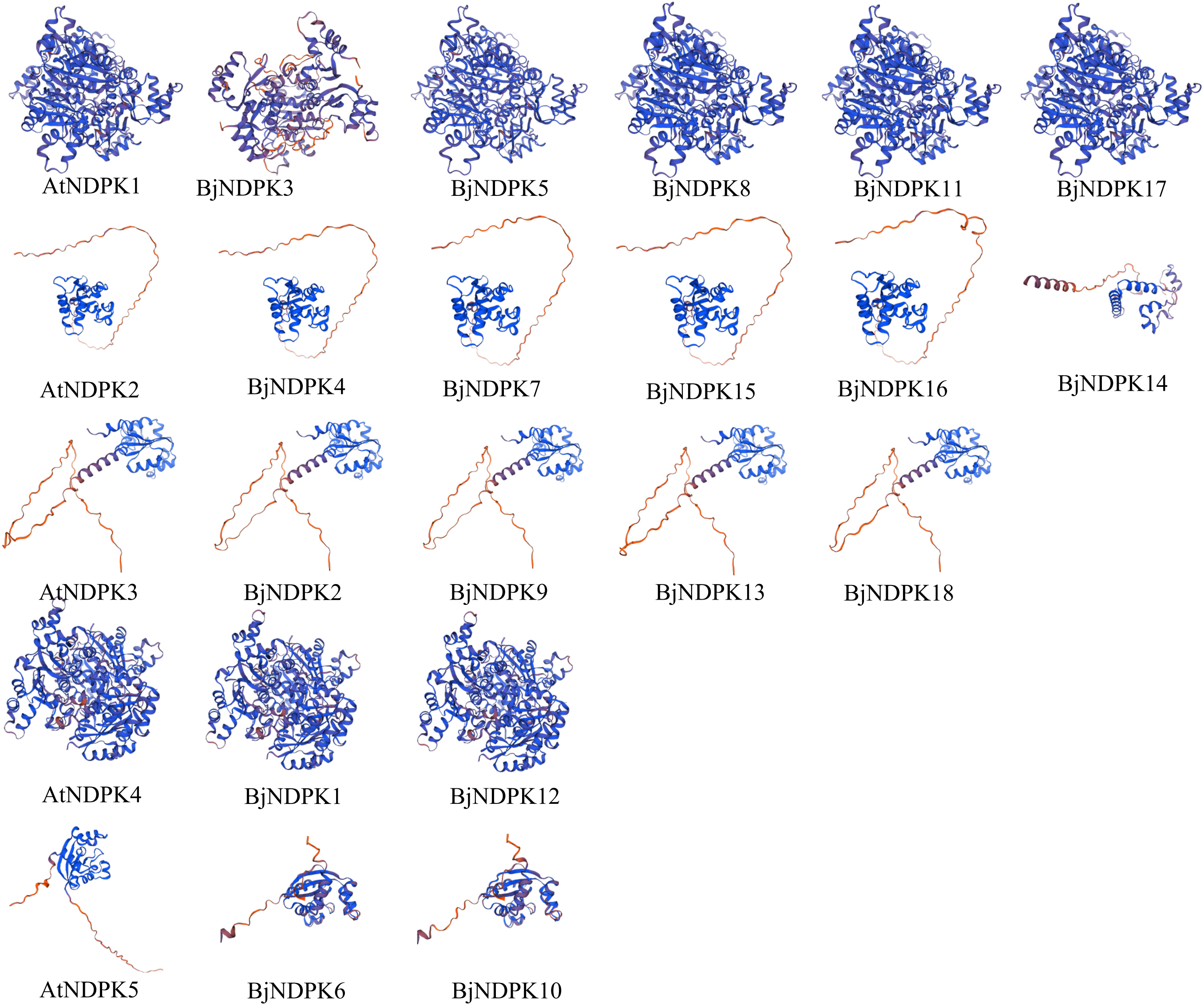
AtNDPK1
BjNDPK3
BjNDPK5
BjNDPK8
BjNDPK11
BjNDPK17
AtNDPK2
BjNDPK4
BjNDPK7
BjNDPK15
BjNDPK16
BjNDPK14
AtNDPK3
BjNDPK2
BjNDPK9
BjNDPK13
BjNDPK18
AtNDPK4
BjNDPK1
BjNDPK12
AtNDPK5
BjNDPK6
BjNDPK10
